# LLM Multimodal Traffic Accident Forecasting

**DOI:** 10.3390/s23229225

**Published:** 2023-11-16

**Authors:** I. de Zarzà, J. de Curtò, Gemma Roig, Carlos T. Calafate

**Affiliations:** 1 Informatik und Mathematik, GOETHE-University Frankfurt am Main, 60323 Frankfurt am Main, Germany; dezarza@em.uni-frankfurt.de (I.d.Z.); roig@cs.uni-frankfurt.de (G.R.); 2 Departamento de Informática de Sistemas y Computadores, Universitat Politècnica de València, 46022 València, Spain; calafate@disca.upv.es; 3 Estudis d’Informàtica, Multimèdia i Telecomunicació, Universitat Oberta de Catalunya, 08018 Barcelona, Spain; 4 HESSIAN Center for AI (hessian.AI), 64289 Darmstadt, Germany

**Keywords:** LLM, VLM, LLaVA, accident forecasting, transformers, time series analysis, PCA loadings

## Abstract

With the rise in traffic congestion in urban centers, predicting accidents has become paramount for city planning and public safety. This work comprehensively studied the efficacy of modern deep learning (DL) methods in forecasting traffic accidents and enhancing Level-4 and Level-5 (L-4 and L-5) driving assistants with actionable visual and language cues. Using a rich dataset detailing accident occurrences, we juxtaposed the Transformer model against traditional time series models like ARIMA and the more recent Prophet model. Additionally, through detailed analysis, we delved deep into feature importance using principal component analysis (PCA) loadings, uncovering key factors contributing to accidents. We introduce the idea of using real-time interventions with large language models (LLMs) in autonomous driving with the use of lightweight compact LLMs like LLaMA-2 and Zephyr-7b-α. Our exploration extends to the realm of multimodality, through the use of Large Language-and-Vision Assistant (LLaVA)—a bridge between visual and linguistic cues by means of a Visual Language Model (VLM)—in conjunction with deep probabilistic reasoning, enhancing the real-time responsiveness of autonomous driving systems. In this study, we elucidate the advantages of employing large multimodal models within DL and deep probabilistic programming for enhancing the performance and usability of time series forecasting and feature weight importance, particularly in a self-driving scenario. This work paves the way for safer, smarter cities, underpinned by data-driven decision making.

## 1. Introduction and Related Work

As urban centers around the world burgeon, the complex web of roads, highways, and streets that weave them together is becoming increasingly congested. With this congestion comes a growing risk: traffic accidents. These accidents, ranging from minor fender benders to major collisions, have significant implications not only for those directly involved but also for the larger community, as they disrupt traffic flow, necessitate emergency response, and, in the worst cases, lead to fatalities.

The ability to predict these accidents, even with a modest degree of accuracy, can be immensely beneficial. For city planners and traffic management agencies, such predictive insights offer a roadmap for the better design of roadways, implementation of safety measures, and planning of traffic routes [1]. Emergency services can optimize their resource allocation, ensuring rapid response times in accident-prone areas [2]. Specifically, our predictions could target both historically accident-prone locations and anticipate high-risk timeframes, allowing for more proactive and precise interventions. For the everyday commuter, understanding accident trends might inform their daily routes, potentially reducing their personal risk, while our methodology primarily targets systemic issues like flawed road designs and historically problematic areas, it also offers insights into dynamic factors that might elevate accident risks, enabling a comprehensive approach to accident prediction and prevention.

Yet, predicting accidents is no trivial task [3,4]. Unlike many other events, the confluence of factors leading to an accident is vast. Weather conditions, time of day, road quality, vehicle density, driver behavior [5], and countless other variables play a role. Traditional approaches to predicting accidents have often revolved around statistical models that, while informative, may not fully encapsulate the intricate interplay of these factors.

With the advent of deep learning (DL) and its proven prowess in handling complex datasets, there is a burgeoning interest in its application to this domain [6,7,8]. If successful, it promises a more nuanced, accurate, and actionable understanding of when and where accidents might occur.

Emphasizing the societal implications [9], leveraging predictive analytics transcends academic pursuits; while the complexity of accident prediction has been elaborated upon, the real-world gains are significant. Harnessing data-driven insights through modern machine learning (ML) techniques [10], such as DL and AI, is not just a technological leap; it is a step towards safer roadways, economic efficiency, and, most importantly, the preservation of human life.

In this work, in addition to proposing the use of principal component analysis (PCA) loadings [11] for more informed choices, that is, the feature weights of the principal components of a given portion of the data under study, we introduced the use of large language models (LLMs) [12,13] as real-time interventors that can modify the driving style of a Level 4 or 5 (L-4, L-5) autonomous driver, take precautions in case of the high probability of a car accident, and act in real-time on an embedded device that works autonomously. Additionally, we integrated the use of visual language models (VLMs) [14] to further increase the response impact of the actionable pipeline [15,16,17] and linked them with deep probabilistic programming [18] for uncertainty quantification.

Contribution:

- Incorporating end-to-end driving into the pipeline of autonomous cars has been at the core of research over the past decades [19]. However, such methodologies have failed to achieve mainstream recognition, and major self-driving companies rely on LiDAR [20] and other sophisticated sensing technologies [21], as well as hard-coded guidelines for specific tasks such as line following. In this paper, we try to partly bridge that gap by proposing a method to modify the driving style of an L-4 or L-5 autonomous vehicle with the use of LLMs and VLMs. For that purpose, we leverage the information of the feature weights of the principal components of PCA, also known as PCA loadings, to help the language models make more informed choices with proper inferred knowledge.
- We advance the use of VLMs directly on visual cues for decision optimization, as this will become mainstream with the appearance of GPT-4V (GPT with Vision).

This is the first work to use PCA feature weights from historical traffic data to help LLMs make informative decisions in real time, combined with real-time image road analysis provided by a large multimodal model, linking explainable AI and LLM motion planning [22,23].

The remainder of this paper is structured as follows: In Section 2, we delineate our research methodology, underlining the key steps and techniques adopted. This is followed by Section 3, where we present our preliminary results, emphasizing the initial findings and patterns discerned. Venturing deeper into the domain, Section 4 delves into the intricate interplay of forecasting, PCA, and the capacity of LLMs within the context of autonomous driving. Section 4.6 extends this dialogue, specifically spotlighting the role of language models in traffic forecasting and real-time road analysis. In Section 5, we introduce LLaVA, a pioneering multimodal understanding model, explicating its unique capabilities and transformative potential in reshaping our understanding of vision and language synthesis. Finally, our study is concluded in Section 6 and Section 7, where we discuss and analyze our insights, reflecting upon the study’s advancements and elucidating the course of future exploration in this novel study area, where we integrate forecasting, language models, and autonomous driving.

## 2. Methods

The dataset used in this study was meticulously sourced from various traffic management systems and contains records spanning several years. Each entry in the dataset represents an accident event and carries with it a plethora of features, from environmental variables like weather conditions, denoted as *W*, to temporal ones such as the time of day, represented by *T*.

(1) $$D=\{\left(W_{1},T_{1}\right),\left(W_{2},T_{2}\right),\ldots ,\left(W_{n},T_{n}\right)\},$$

where *D* is the dataset and *n* is the total number of records.

The raw data, while comprehensive, required significant preprocessing to be suitable for ML models. The primary steps involved:

- Normalization: Features with varying scales were normalized using the standard Z-score formula:
  (2) $$Z=\frac{X-\mu }{\sigma },$$
  where *X* is the feature value, μ is the mean, and σ is the standard deviation.
- Handling Missing Values: Missing values, represented as ∅, were imputed using mean imputation.
  (3) $$X_{o}=\left\{\begin{array}{cc} X_{o} & \mathrm{if}\;X_{o}\neq \emptyset ,\\ \mu & \mathrm{otherwise}. \end{array}\right.$$
- Feature Encoding: Categorical variables were converted into numerical ones using one-hot encoding, expanding the feature space from *m* dimensions to *k* dimensions.

The datasets hail from the Fatality Analysis Reporting System (FARS), 2020, which is a comprehensive repository detailing accidents across the USA. Specifically, the *accidents.csv* file from the 2020 National CSV archive was employed for this study, data can be found at: https://static.nhtsa.gov/nhtsa/downloads/FARS/2020/National/FARS2020NationalCSV.zip (accessed on 1 October 2023). This dataset offers a vast array of features encompassing the specifics of each accident, including details related to time, location, vehicles involved, and the conditions under which the accident occurred.

An initial analysis was conducted to grasp the nature and structure of the data. Our investigation first centered on comprehending the different attributes and their types, followed by identifying and handling missing or malformed data. Recognizing and addressing these issues is paramount to ensure the integrity and reliability of subsequent analyses.

Driven by both the dataset’s documentation and domain-specific knowledge, we identified a subset of the dataset deemed most relevant for our analysis. This subset encompasses attributes related to fatalities, influence of alcohol, total vehicles involved, geographical location, time of occurrence, events causing harm, and prevailing weather conditions, among others.

**Figure 1:**
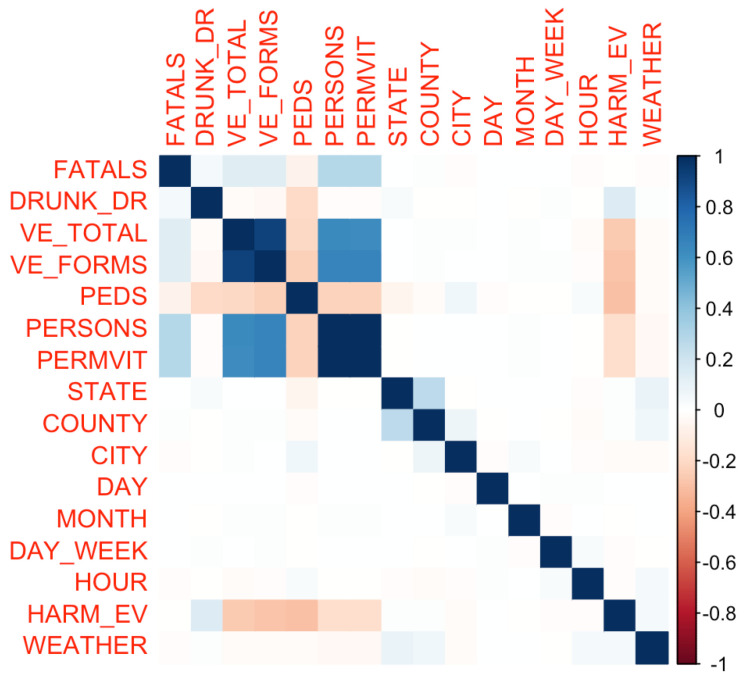
Correlation matrix visualization: This plot provides a visual representation of the pairwise correlation between the selected numeric attributes from the accident dataset. The color intensity and the size of the circles correspond to the correlation coefficients. Positive correlations are displayed in blue and negative correlations in red. This visualization aids in identifying attributes that might have a strong association with each other, providing insights into potential multicollinearity and relationships within the data.

After feature selection, a correlation matrix was constructed to gauge the interdependencies between the various numerical attributes, as can be observed in Figure 1. The correlation analysis aids in discerning patterns and relationships inherent in the dataset, offering preliminary insights that can guide subsequent analyses.

Explanation of Variables:

- FATALS represents the number of fatalities that occurred in a given accident. It provides insights into the severity of an accident and helps prioritize interventions for high-risk scenarios.
- DRUNK_DR indicates the number of drivers involved in the fatal crash who were under the influence of alcohol. It is a key metric in understanding the impact of impaired driving on road safety.
- VE_TOTAL represents the total number of vehicles involved in the crash. This can help identify multi-vehicle pile-ups or accidents involving multiple parties.
- VE_FORMS refers to the different forms or types of vehicles involved in the accident. This can distinguish between accidents involving, for instance, passenger cars, trucks, or motorcycles.
- PEDS indicates the number of pedestrians involved in the accident. Pedestrian safety is a critical concern in urban areas, and this metric can help in understanding risks to those not in vehicles.
- PERSONS represents the total number of individuals involved in the crash, both vehicle occupants and pedestrians.
- PERMVIT refers to the number of person forms submitted for a given accident. It provides a count of how many individual records or forms were documented for the incident.
- STATE is the state in which the accident occurred. Geographic segmentation can help identify regional trends or areas with higher accident rates.
- COUNTY specifies the county of the accident location. This finer geographic detail can assist in local interventions and in understanding accident hotspots at a county level.
- CITY represents the city where the accident took place. Urban areas might have different accident patterns compared to rural regions.
- DAY is the day of the month on which the accident occurred, which can help identify specific days with higher accident rates.
- MONTH is the month of the year in which the accident took place. Seasonal trends, like winter months having more accidents due to slippery roads, can be analyzed using this metric.
- YEAR is the year of the accident, crucial for trend analysis over multiple years and understanding long-term changes in accident patterns.
- DAY_WEEK is the day of the week on which the accident occurred. Some days, like weekends, might have different accident patterns due to increased travel or recreational activities.
- HOUR is the hour of the day when the accident happened. This can provide insights into rush hour patterns, nighttime driving, etc.
- HARM_EV represents the first harmful event or the initial injury or damage-producing occurrence in the accident. It sheds light on the primary cause or event leading to the accident.
- WEATHER describes the atmospheric conditions at the time of the accident. Weather factors significantly into driving conditions, and this metric can elucidate accidents related to rain, fog, snow, etc.

While the raw dataset is very useful, its true potential is often unearthed when supplemented with engineered features. In our study, we introduced a new attribute, “time_of_day”, derived from the “HOUR” feature. This addition aims to offer a clearer understanding of when accidents predominantly occur, segmenting the day into intuitive time intervals.

**Figure 2:**
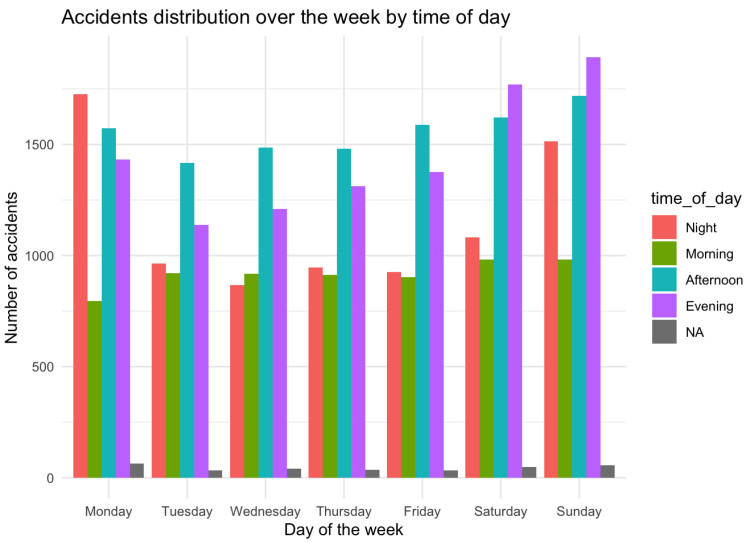
Accidents distribution over the week by time of day.

A graphical representation in Figure 2 was crafted to depict the distribution of accidents over the week, segmented by the time of day. This visualization serves as a compelling illustration of accident frequencies, facilitating the easy discernment of peak accident-prone periods.

- Initialization of Libraries and Data Loading: First, we loaded the essential libraries, ensuring they were installed, to set up our working environment. Following this, the dataset titled "accident.csv" was imported into the R environment for subsequent analyses.
- Data Structure and Preliminary Analysis: A fundamental understanding of the dataset’s structure is vital. Using the str() function, we observed the dimensions and variable types and viewed the initial entries of the dataset. To ensure data integrity, we searched for missing values and empty strings, laying the groundwork for any necessary preprocessing.
- Feature Selection: A subset of relevant variables was selected, influenced by prior domain knowledge and the analytical goals.
- Correlation Analysis: A correlation matrix was created to understand the relationships between numerical attributes, highlighting any potential interdependencies or redundancies.
- Summary of Accident Data Analysis Results
  - Preliminary Analysis: The dataset includes various attributes, such as information on the number of vehicles involved, alcohol influence, weather conditions, and the time of occurrence. Missing values in some columns were addressed during the analysis.
  - Key Data
    * On average, there are approximately 1.085 fatalities per accident, with a maximum of 8 fatalities in a single accident.
    * Approximately 26.6% of accidents involve a drunk driver.
    * Most accidents involve between one and two vehicles, and about 23% of accidents involve pedestrians.
    * Accidents are distributed throughout the day, with peaks in the afternoon and evening.
  - Correlation Analysis: VE_TOTAL and VE_FORMS are highly correlated. Accidents involving pedestrians (PEDS) tend to have fewer vehicles and persons.
  - Temporal Information: Accidents primarily occur in the afternoon and evening, with noticeable increases on certain weekdays.

In our methodology, we leveraged a variety of models, including the Transformer [24], ARIMA [25], and Prophet [26], to forecast traffic accidents using time series data. To prepare the data for the Transformer architecture, we first converted our time series data into a supervised learning format. This conversion allows us to utilize past observations as input variables to predict the next time step. The transformation process facilitates this by creating a structured dataset with columns representing the current time step and any preceding time steps that might be used as input. Given the sequential nature of our data, the data were standardized to ensure that all values lay between 0 and 1. This normalization process facilitates better convergence during the training phase and enhances the overall performance of DL models.

To adapt the data for supervised learning, we transformed our time series into a lagged format using a custom function. This function constructs a DataFrame where, for each current time step, preceding time steps are added as additional columns, making it possible to predict the future time step based on prior observations. In essence, it shifts the series to create input–output pairs where past values (lag values) act as the input and the current value acts as the output. This format is conducive for neural network architectures which require a clear demarcation between input features and target output.

After normalization, our dataset was restructured to align with the requirements of specific layers within our Transformer architecture. To carve out a training and testing landscape, 80% of the data were designated for training, with the remainder reserved for validation. Keeping the sequential nature of the data intact, this split was performed chronologically. Each dataset was then segregated into input and output segments, and the input data were reshaped to be compatible with the three-dimensional input construct expected by certain layers: samples, time steps, and features.

In subsequent steps, the data were supplied to our selected models—Transformer, ARIMA, and Prophet. Each model was then calibrated using the training dataset, and its forecasting capabilities were assessed using the validation dataset. By comparing the performance metrics of these models, we strive to identify the most efficient approach for our traffic accident forecasting endeavors.

ARIMA [27], which stands for AutoRegressive Integrated Moving Average, is a time series forecasting model that combines autoregressive (AR) and moving average (MA) approaches. The equation for ARIMA can be denoted as:

(4) $$Y_{t}=\alpha +\beta_{1}Y_{t-1}+\cdots +\beta_{p}Y_{t-p}+\theta_{1}\epsilon_{t-1}+\cdots +\theta_{q}\epsilon_{t-q}+\epsilon_{t},$$

where $Y_{t}$ is the value at time *t*; β and θ are the AR and MA coefficients, respectively; and ϵ is the error term.

Prophet [28,29], developed by Facebook, is a forecasting tool designed for daily datasets that exhibit strong seasonal patterns. It accounts for holidays and allows the addition of custom seasonalities, making it robust for various applications.

Transformers [30] leverage attention mechanisms, enabling them to focus on different parts of the input data, making them highly effective for sequence data. The attention in the Transformers is calculated as:

(5) $$\mathrm{Attention}\left(Q,K,V\right)=\mathrm{softmax}\left(\frac{QK^{T}}{\sqrt{d_{k}}}\right)V,$$

where *Q*, *K*, and *V* are the query, key, and value matrices, respectively, and $d_{k}$ is the dimensionality of the queries.

Delving into our specific instantiation of the Transformer architecture, we harnessed TensorFlow’s Keras API. The Transformer model’s core component is its encoder, which is constructed with multi-head attention followed by position-wise feed-forward networks. In our setup,

- Head Size: The size of each head in the multi-head attention mechanism is set to 256.
- Number of Heads: We employ four attention heads, allowing the model to focus on different parts of the input simultaneously.
- Feed-forward Dimension: The dimensionality of the feed-forward network’s inner layer is set to 4.
- Number of Transformer Blocks: Our model is equipped with four Transformer blocks, each containing one multi-head attention layer and one feed-forward layer.
- MLP Units: After the Transformer blocks, the sequence is pooled globally and then passed through a feed-forward network with 128 units.
- Dropout: Dropout layers with rates of 0.4 (for the MLP) and 0.25 (for the Transformer encoder) are integrated to mitigate overfitting.

The constructed model was trained using the mean squared error loss with the Adam optimizer. The training process spans 50 epochs, utilizing a batch size of 8.

Singular value decomposition (SVD) is a matrix factorization technique that decomposes a matrix *A* into three matrices *U*, Σ, and $V^{T}$. It can be represented as follows:

(6) $$A=U\Sigma V^{T}.$$

In the context of feature importance, the matrix $V^{T}$ provides an indication of the significance of each feature in the dataset. The loadings of features in $V^{T}$ can be used to rank and discern the most influential features in predicting outcomes.

## 3. Evaluation

**Figure 3:**
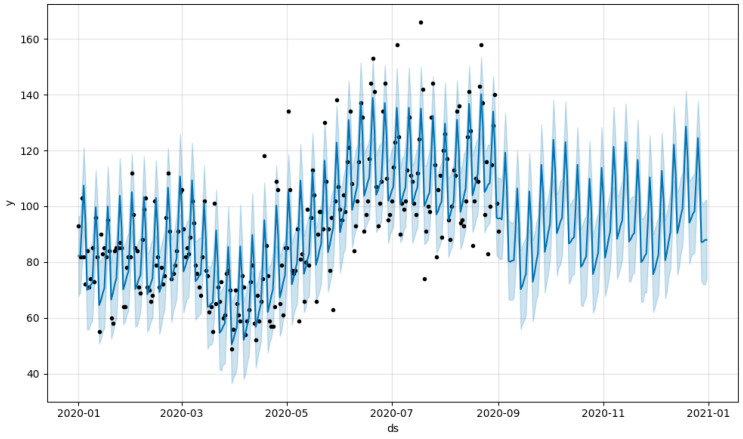
Model forecast with Prophet, with daily and yearly seasonality. The x-axis represents the date. The y-axis denotes the number of accidents. The dark dots represent the actual historical data of accidents, while the light blue line illustrates the forecasted values. The shaded blue region represents the uncertainty intervals of the forecast.

**Figure 4:**
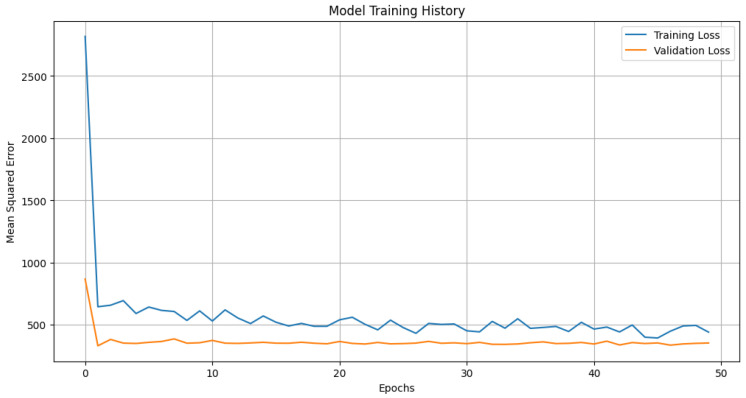
Model training and validation loss for the Transformer architecture.

**Figure 5:**
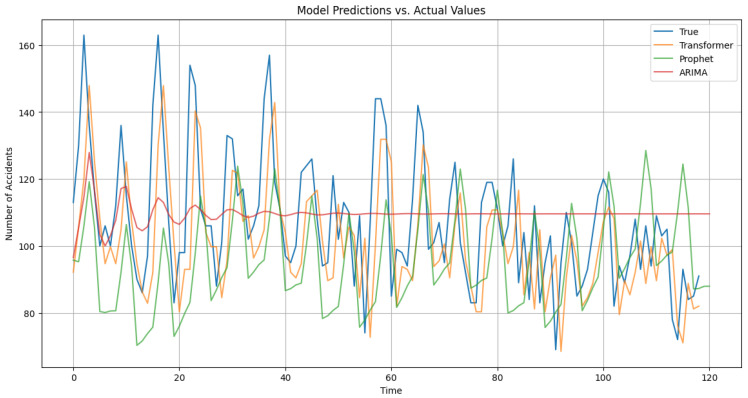
Comparison of model predictions against actual values.

Figure 3 illustrates the model predictions for Prophet, with daily and yearly seasonality. Figure 4 depicts training and validation loss for the Transformer architecture under study. Figure 5 showcases the predictions from our Transformer model juxtaposed against the actual daily accident counts in comparison with Prophet and ARIMA. These actual values represent the true number of traffic accidents recorded each day. The plot provides a visual representation of the model’s approximation to this real-world daily accident data.

### 3.1. Detailed Exploration of Top Feature Weights

To shed light on the most influential features driving accident occurrences, an analysis using singular value decomposition (SVD) was performed. The loadings from the matrix $V^{T}$ in the SVD decomposition were extracted and ranked.

**Figure 6:**
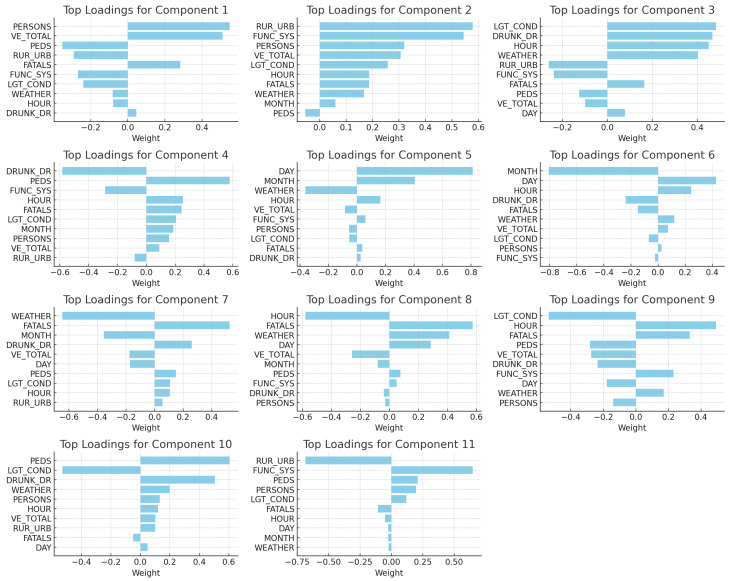
Top feature weights across different components of the PCA loadings.

Figure 6 illustrates the top feature weights across different components of the decomposition.

#### Implications of Top Feature Weights

- Trafficway Context: Features related to the type and classification of the trafficway, such as non-state inventoried roadways, played a significant role in accident occurrences. This indicates the importance of infrastructural factors in determining accident risk.
- Light and Environmental Conditions: Variables related to lighting (e.g., dawn or dusk) and weather conditions (e.g., cloudy) were paramount, suggesting that visibility and environmental factors substantially influence the likelihood of accidents.
- Temporal Factors: Features related to specific times, such as early mornings or specific days in a month, were notable, emphasizing the periodic nature of accident risks.

These insights highlight the multifaceted nature of accident risks, with a confluence of infrastructural, environmental, and temporal factors at play [31]. The Transformer architecture, originally designed for natural language processing tasks, has demonstrated efficacy in our study for time series forecasting of daily accidents. Its ability to consider patterns, dependencies, and temporal sequences in the data concurrently enabled it to outperform traditional models.

### 3.2. Interpretation of PCA Loading Results

The SVD offers a glimpse into the feature weights and their consequential importance in accident predictions.

*Component Analysis*: The PCA loadings highlight distinct characteristics for each of the 11 components. Component 1 reveals significant influence from factors like trafficways not affiliated with the state inventory, unknown lighting during the crash, and the involvement of drivers under the influence of alcohol (see Table 1). Component 2 is characterized by the number of vehicles and individuals involved in the crash, lighting conditions at dawn, fatalities, and cloudy weather conditions. Similarly, Component 3 emphasizes the roadway’s functional system, land use patterns, the number of fatalities, and specific accident timings. Component 4 predominantly focuses on lighting conditions, the urban nature of the crash site, and the roadway’s system (see Table 2). Component 5 offers insights into the month of the accident, the number of involved persons, and the crash’s urban or rural context. Component 6 draws attention to the number of involved vehicles, especially on interstate roads, and the total number of involved individuals.

Component 7 highlights influences from the functional system of the roadway, vehicle counts, and specific times of accidents. Component 8 is distinguished by its emphasis on the functional system of the road, the count of non-motorists, and the prevailing weather during the crash. Component 9, on the other hand, emphasizes the number of involved individuals, specific accident dates, and the functional system of the road. Component 10 stresses the roadway’s functional system, specific accident dates, and the accident hour. Lastly, Component 11 showcases strong correlations with non-motorist counts during the crash, urbanization indications of the crash site, and the functional system of the roadway.

A detailed description of these loadings is further elaborated in Table 1, Table 2 and Table 3.

These derived components, essentially linear combinations of the initial features, distinctly portray multifaceted scenarios and accident risk factors.

### 3.3. Implications of the Findings

The Transformer model’s good prediction in its ability to rapidly adapt to the curve fluctuations and the ARIMA moving average, as presented in Figure 5, augurs well for future safety protocols and urban developmental strategies that could be used as seed information for detailed LLM forecasting. As such, accurate forecasts can spearhead resource allocation, especially in zones or temporal phases predicted to witness elevated accident frequencies.

Furthermore, the SVD’s hints underscore the multifaceted origins of accidents. While some determinants like nebulous lighting conditions or the influence of alcohol are universally acknowledged accident harbingers, the nuanced patterns of the SVD components advocate for a comprehensive approach to accident prevention. The essence is not merely about amplifying road quality or instituting stringent driving regulations, it is about understanding the many determinants that coalesce in real-world scenarios.

Moreover, this exploration underlines the imperative to perpetually advance and adopt smart predictive models for such endeavors. As urban landscapes burgeon in complexity and population, a precise forecasting instrument has become a sine qua non condition for urban strategists, policy architects, and public safety custodians.

## 4. The Interplay of Forecasting, PCA, and Large Language Models in Autonomous Driving

In the quest for safer roads, the coupling of state-of-the-art ML methods, such as model forecasting, with classical techniques, like principal component analysis (PCA), holds promise. However, the synthesis of these methods finds its true potential when combined with the linguistic capabilities of large language models (LLMs) [24,32].

### 4.1. Formalizing Accident Prediction for Autonomous Driving

To frame the problem, let *X* represent our data matrix, where each row corresponds to a different time or location, and where each column captures a relevant feature (e.g., weather condition, traffic volume). Our goal is to forecast *y*, the likelihood of an accident occurring.

The Transformer-based forecasting model can be defined as follows:

(7) f:X→y,

where *f* is our forecasting function. Given an input data matrix *X*, it predicts *y*, the future accident likelihood.

### 4.2. Incorporating Principal Component Analysis

While *X* might contain rich information, it could also be rife with redundancies. To capture the essence of our data, we employ PCA, a dimensionality reduction technique. PCA identifies linear combinations (principal components) of the columns of *X* that capture the maximum variance in the data.

Given *X*, the first principal component is defined as follows:

(8) $$c_{1}=\underset{c}{\arg\max}\{\mathrm{Var}\left(X_{c}\right){:||c||}_{2}=1\}.$$

Subsequent principal components $c_{2},c_{3},\ldots$ are orthogonal to $c_{1}$ and capture the remaining variance in decreasing order.

These principal components, especially the leading ones, encapsulate the most significant factors affecting accident likelihood. These factors, when combined with model forecasts, offer a potent tool for risk assessment.

The current generation of autonomous vehicles (especially L-4 and L-5) rely heavily on sensor data, pre-set algorithms, and predefined safety protocols rather than dynamically adapting based on text-based insights.

However, our intention with the presented framework is not to suggest that the vehicle would directly parse and comprehend the text. Instead, the text-based insights are meant for human experts to interpret and subsequently refine the algorithms that guide the vehicle’s behavior. Once these refined algorithms are tested and verified, they can be pushed as updates to the vehicle’s decision-making system.

For example, if our model consistently predicts higher accidents during certain lighting conditions combined with specific traffic patterns, the driving algorithms can be adjusted to make the vehicle more cautious during those conditions. The suggestions provided in the textual insights, such as slowing down during low light or being more vigilant in areas with unknown land use, can be translated into tangible algorithmic adjustments.

Furthermore, as the field of autonomous driving evolves, there is the potential to integrate more advanced AI models, as we present in the subsequent sections with the use of LLMs and VLMs, to directly influence the decision-making process in real-time. This would require a seamless integration where the AI’s predictions and recommendations are automatically translated into decision parameters for the autonomous system.

### 4.3. Leveraging Large Language Models in Autonomous Driving

While numerical models excel in analyzing structured data, they falter when confronted with unstructured data like natural language. Here, LLMs [33] come to the fore. Equipped with an immense understanding of language, LLMs can discern intricate patterns and nuances in textual data.

Consider an autonomous vehicle equipped with a communication system receiving real-time feedback, road reports, or even social media snippets hinting at road conditions. LLMs can process this unstructured data, converting linguistic patterns into risk factors, which we denote as $R_{LLM}$.

The combined risk, incorporating model forecasts and LLM insights, is thenas follows:

(9) $$R_{total}=f\left(Xc\right)+\alpha R_{LLM},$$

where α is a weighting factor.

### 4.4. Real-Time Intervention for Safer Autonomous Driving

For autonomous vehicles, particularly those of Levels 4 and 5 (L-4, L-5), the ability to assess and adapt to risks in real-time is paramount. Using the combined risk metric $R_{total}$, the vehicle’s driving system can adjust its conduction style dynamically. High-risk scenarios might trigger more conservative driving, while low-risk scenarios allow for normal operations.

To formalize, the driving style *S* at any time *t* can be expressed as a function of the risk:

(10) $$S\left(t\right)=g(R_{total}\left(t\right)),$$

where *g* is a predefined function mapping risk levels to driving styles.

The fusion of DL forecasting, PCA, and LLMs offers a multifaceted approach to enhancing the safety protocols of autonomous vehicles. In a world transitioning to autonomous driving, ensuring the highest safety standards is not just a technological challenge but also a moral imperative. Through rigorous research and the application of advanced ML methodologies, we move closer to a future where roads are safer for everyone.

### 4.5. Zephyr-7b-α in Autonomous Driving: Pioneering Real-Time Interventions

Zephyr-7b-α, a derivative of the model Mistral [34,35], has been meticulously fine-tuned for specific applications by leveraging datasets like Ultrachat and Ultrafeedback, focusing on conversational abilities. Recognizing the sheer potential of this specialized model, we contemplate its utility in the realm of autonomous driving, specifically for real-time risk interventions.

From Mistral to Zephyr-7b-α in autonomous driving: Traditional approaches to autonomous driving have been largely based on sensory data. However, the dynamic nature of road environments requires a deeper understanding. Zephyr-7b-α, with its advanced conversational capabilities, can process natural language information in real time—be it traffic updates, reports of incidents ahead, or even conversational data from nearby vehicles. This offers an added layer of intelligence, aiding the vehicle in comprehending its surroundings beyond mere sensory input.

Building upon Mistral’s efficiency, Zephyr-7b-α introduces an enriched context sensitivity essential for real-time driving scenarios. For instance, while sensory data might indicate clear roads ahead, Zephyr-7b-α can process live traffic updates and recognize a potential congestion situation a few miles ahead, enabling the vehicle to make proactive decisions.

Real-time risk assessment and mitigation: Employing LLMs like Zephyr-7b-α can dramatically enhance the real-time risk assessment capabilities of autonomous vehicles. By continuously integrating real-time linguistic data with traditional sensory inputs and the Transformer-based forecasting models, a comprehensive risk profile can be developed. Mathematically, the risk factor *R* at any given moment *t* could be expressed as follows:

(11) $$R\left(t\right)=f_{forecast}\left(X_{sensory}\right)+\beta f_{LLM}\left(X_{linguistic}\right),$$

where $f_{forecast}$ is the risk predicted by the Transformer model based on sensory data $X_{sensory}$, $f_{LLM}$ represents the risk assessment from Zephyr-7b-α based on linguistic data $X_{linguistic}$, and β is a weighting factor.

As the vehicle navigates, Zephyr-7b-α continuously adjusts its risk profiles. Elevated risk levels can trigger adaptive driving mechanisms, allowing the vehicle to preemptively adjust its speed, lane, or even the route.

On-the-Edge Processing: One of the crowning features of Zephyr-7b-α is its compact design, catering to applications where computational resources are a constraint. This makes it particularly suitable for on-the-edge processing in autonomous vehicles; while cloud-based processing offers extensive computational power, the real-time demands of driving and concerns over network latency emphasize the need for localized processing. Zephyr-7b-α, with its balance between model size and capability, has emerged as an ideal candidate.

For instance, an example query is shown in Box 1.

Box 1 **LLM Autonomous Driving System Interaction.**

messages = [ { "role": "system", "content": "You are an autonomous driving expert and safety analyst. You are familiar with forecasting models, accident prediction, and traffic data interpretations." }, { "role": "user", "content": f"On the given day, our Transformer model predicts {predicted_accidents} accidents. The PCA loadings for the day’s features are as follows: {loadings_string}. Given this information, how should an autonomous vehicle adjust its driving behavior in real-time to minimize risk?" } ]

The integration of Zephyr-7b-α into the autonomous driving ecosystem exemplifies the convergence of cutting-edge technologies for groundbreaking applications. By synthesizing real-time linguistic processing with traditional risk forecasting, we pave the way for a new generation of intelligent, proactive, and safer autonomous vehicles. As technology continues to evolve, models like Zephyr-7b-α underscore the limitless possibilities at the intersection of language processing and autonomous systems.

### 4.6. Utilizing Language Models for Traffic Forecasting and Analysis

In the recent era of AI, the use of pre-trained language models [36,37] has garnered significant attention, not just for natural language processing tasks but also for a plethora of diverse applications. In our research, we have incorporated the power of three distinct models, OpenAI’s GPT-4, LLaMA-2, and Zephyr-7b-α, to delve into PCA loading interpretation and advanced traffic forecasting. Furthermore, LLaVA-13b [38], a state-of-the-art visual language model, was utilized for intricate image road analyses, as outlined in Section 5.

#### OpenAI GPT-4, LLaMA-2, and Zephyr-7b-α in Traffic Forecasting

The principal component analysis (PCA) loadings provide a compact representation of the most significant patterns in our traffic dataset. However, understanding and interpreting these loadings can be challenging. This is where pre-trained language models come into play. OpenAI’s GPT-4, a latest-generation model known for its incredible probabilistic text generation capabilities, assists in providing intuitive insights into these PCA loadings. By feeding the model with the PCA loadings, we extract human-understandable patterns and relationships embedded within the traffic data. LLM prompt and PCA Loadings analysis with OpenAI ’gpt4-0613’ is shown in Box 2 and Box 3.

LLaMA-2, another noteworthy model, provides similar capabilities but with distinct underlying mechanics and architecture. The differentiation in the approach of LLaMA-2 offers an alternative interpretation which, when combined with GPT-4’s insights, delivers a comprehensive understanding of the traffic patterns. LLM prompt and PCA Loadings analysis with Meta ’LLaMA-2-13b-chat-hf’ is shown in Box 4 and Box 5.

Zephyr-7b-α, on the other hand, specializes in real-time responses. By leveraging its high-speed processing capabilities, we derive real-time traffic forecasts that not only predict potential congestions but also assist in identifying optimal routes and navigation strategies. LLM prompt and PCA Loadings analysis with ’HuggingFaceH4/zephyr-7b-alpha’ is shown in Box 6 and Box 7.

Box 2 **LLM prompt with OpenAI model ’gpt4-0613’.**

response = openai.ChatCompletion.create( model=“gpt-4-0613”, messages=[ { “role”: “system”, “content”: “You are an autonomous driving expert and safety analyst. You are familiar with forecasting models, accident prediction, and traffic data interpretations”. }, { “role”: “user”, “content”: f”On the given day, our Transformer model predicts {predicted_accidents} accidents. The PCA loadings for the day’s features are as follows:{loadings_string}Given this information, how should an autonomous vehicle adjust its driving behavior in real-time to minimize risk?” } ] )

Box 3 **LLM PCA Loadings analysis with OpenAI model ’gpt4-0613’.**

Based on the PCA loadings of your forecast model, an autonomous vehicle can take into account certain behaviors to minimize the risk of accidents. Here are a few strategies:

1. Time of Day: Many components heavily factor in the time of day, with high accident risk during early morning hours (6 a.m., 7 a.m., 8 a.m.) and late evening hours (5 p.m., 7 p.m., 8 p.m.). During these hours, the autonomous vehicle can adopt a more cautious driving profile, maintaining slower speeds and increasing distance from other vehicles.

2. Lighting Conditions: Accidents seem to occur more often when the lighting conditions are not optimal, such as “Dark but Lighted”, “Dark – Not Lighted”, and Dusk. The vehicle can increase the usage of its headlights, lidar, or other night-vision technologies to improve visibility.

3. Unknown Variables: Incidents happen more frequently when variables such as “Land Use” or “Functional System” are unknown. The autonomous vehicle could use real-time tracking to constantly update its knowledge of traffic conditions, weather, and the environment.

4. Weather Conditions: Weather conditions like fog, smog, smoke, and cloudy weather seem to contribute to the likelihood of accidents. The autonomous vehicle should leverage its weather detection sensors to adjust its speed, braking distance, and alertness level during poor weather conditions.

5. Multiple Persons/Vehicles Involvement: A notable number of accidents recorded involve two vehicles or crashes where multiple persons are involved. The vehicle should prepare for sudden maneuvers in traffic-rich or pedestrian-rich environments.

6. Interaction with Drunk Drivers: Component 1 shows that if there is a drunk driver involved, there is a higher risk of an accident. Autonomous vehicles should employ defensive-driving tactics to accommodate unpredictable behavior from other drivers.

Box 4 **LLM prompt with ’LLaMA-2-13b-chat-hf’.**

prompt = f”You are an autonomous driving expert and safety analyst. You are familiar with forecasting models, accident prediction, and traffic data interpretations. On the given day, our Transformer model predicts {predicted_accidents} accidents. The PCA loadings for the day’s features are as follows:{loadings_string}Given this information, how should an autonomous vehicle adjust its driving behavior in real-time to minimize risk?”

Box 5 **LLM PCA Loadings Analysis with ’LLaMA-2-13b-chat-hf’.**

From the given information, we can identify the following factors that contribute to the risk of accidents: 1. Lighting conditions: The presence of darkness or dusk, and the absence of lighted conditions, increases the risk of accidents. 2. Traffic conditions: The presence of two vehicles in the crash, and the absence of pedestrians, suggests that traffic conditions may have contributed to the risk of accidents. 3. Time of day: The accidents occurred between 5 p.m. and 11 p.m., which suggests that the risk of accidents may be higher during these hours. 4. Land use: The presence of unknown land use in some of the components suggests that the risk of accidents may be higher in areas with unknown land use.

Based on these factors, an autonomous vehicle can adjust its driving behavior in real-time to minimize the risk of accidents by adopting the following:

1. Slowing down: The autonomous vehicle can slow down in areas with low lighting conditions or unknown land use to reduce the risk of accidents.

2. Avoiding peak traffic hours: The autonomous vehicle can avoid traveling during peak traffic hours (5 p.m. to 11 p.m.) to reduce the risk of accidents.

3. Increasing vigilance: The autonomous vehicle can increase its vigilance in areas with low lighting conditions or unknown land use to detect potential hazards and take appropriate action.

4. Using advanced sensors: The autonomous vehicle can use advanced sensors such as lidar, radar, and cameras to detect potential hazards and take appropriate action.

5. Communicating with other vehicles: The autonomous vehicle can communicate with other vehicles in the area to exchange information about traffic conditions and potential hazards. By adjusting its driving behavior in real-time based on these factors, an autonomous vehicle can minimize the risk of accidents and improve safety on the road.

Box 6 **LLM prompt with ’HuggingFaceH4/zephyr-7b-alpha’.**

messages = [ { “role”: “system”, “content”: “You are an autonomous driving expert and safety analyst. You are familiar with forecasting models, accident prediction, and traffic data interpretations”. }, { “role”: “user”, “content”: f”On the given day, our Transformer model predicts {predicted_accidents} accidents. The PCA loadings for the day’s features are as follows:{loadings_string}Given this information, how should an autonomous vehicle adjust its driving behavior in real-time to minimize risk?” } ]

Box 7 **LLM PCA Loadings Analysis with ’HuggingFaceH4/zephyr-7b-alpha’.**

Based on the given PCA loadings, the autonomous vehicle should adjust its driving behavior in real-time by taking the following measures to minimize risk:

1. Pay attention to the time of day: The loadings suggest that accidents are more likely to occur during darker hours of the day, particularly in the evening (HOUR_20, HOUR_17, HOUR_18, HOUR_23, HOUR_0) and early morning (HOUR_5, HOUR_6, HOUR_7). Therefore, the vehicle should activate its headlights and high beams when it is dark outside, and use its sensors to detect pedestrians and other vehicles in low-light conditions.

2. Be cautious in rural or urban areas: The loadings suggest that accidents are more likely to occur in areas that are not in the state inventory (FUNC_SYS_96, RUR_URB_6, RUR_URB_9) or have unknown land use (RUR_URB_9). Therefore, the vehicle should slow down and proceed with caution in these areas.

3. Be aware of weather conditions: The loadings suggest that accidents are more likely to occur in fog, smog, or smoke (WEATHER_5). Therefore, the vehicle should adjust its speed and use its sensors to detect any obstacles or hazards in these conditions.

4. Watch out for pedestrians: The loadings suggest that accidents are more likely to involve pedestrians (PEDS_1, PEDS_1). Therefore, the vehicle should use its sensors to detect pedestrians and slow down or stop if necessary.

5. Be alert for drunk drivers: The loadings suggest that accidents are more likely to involve drunk drivers (DRUNK_DR_1). Therefore, the vehicle should be on high alert for signs of erratic driving and report any suspected drunk drivers to the authorities.

6. Be cautious during dusk: The loadings suggest that accidents are more likely to occur during dusk (LGT_COND_5). Therefore, the vehicle should use its headlights and high beams and proceed with caution during this time.

7. Keep an eye out for intersections: The loadings suggest that accidents are more likely to occur during the day (DAY_1, DAY_5, DAY_6, DAY_14, DAY_15, DAY_28, DAY_30, DAY_31) and particularly during the first or second day of the month (DAY_1, DAY_2). Therefore, the vehicle should be on high alert for intersections and proceed with caution.

8. Watch for fatal accidents: The loadings suggest that fatal accidents are more likely to occur (FATALS_2, FATALS_4). Therefore, the vehicle should be on high alert for signs of a fatal accident and proceed with caution.

9. Be cautious during rush hour: The loadings suggest that accidents are more likely to occur during rush hour (HOUR_8). Therefore, the vehicle should be on high alert for signs of traffic congestion and proceed with caution.

10. Be aware of the number of persons and vehicles involved: The loadings suggest that accidents are more likely to involve multiple persons or vehicles (PERSONS_2, VE_TOTAL_2). Therefore, the vehicle should be on high alert for signs of a multi-vehicle accident and proceed with caution. Overall, the autonomous vehicle should use its sensors and real-time data to adjust its driving behavior and minimize risk based on the given PCA loadings.

## 5. LLaVA: A Pioneer in Multi-Modal Understanding for Image Road Analysis

The essence of human cognition lies in our multi-faceted interaction with the world, most notably through vision and language. Each medium has its distinct strengths in capturing and conveying specific aspects of reality, ultimately enriching our comprehension of our surroundings.

Artificial intelligence aims to emulate this profound interplay. The dream is a versatile assistant capable of assimilating both visual and linguistic cues, aligning seamlessly with human intentions, and executing tasks in real-world contexts. To this ambitious end, the AI research community has fervently pursued foundational models that meld language with vision.

While many strides have been made in the arena of vision-focused models, they often function in isolated capacities, each tailored for a specific task, and largely rely on language as a mere descriptor of visual content. This approach, although effective, caps the fluidity and adaptability these models can offer.

On the other end of the spectrum, LLMs like ChatGPT and GPT-4 have championed the use of language as a dynamic, all-encompassing interface. Rather than being confined to predefined roles, these LLMs adapt on-the-fly, interpreting a wide range of task instructions embedded within the language. Notably, the LLaMA model stands out, matching even the prowess of renowned models like GPT-3. Models like Alpaca and Vicuna further amplify the potential of LLMs by integrating high-caliber, machine-generated samples, ensuring unparalleled alignment with user instructions. However, a significant caveat to these strides is the predominantly text-centric nature of the models.

This backdrop sets the stage for Large Language and Vision Assistant (LLaVA), similar to the recent appearance of GPT-4V. LLaVA heralds the dawn of “visual instruction-tuning”, extending the realms of instruction-tuning to encompass both vision and language, laying the foundational stones for an all-encompassing visual assistant. The LLaVA framework, at its core, combines the visual prowess of models like CLIP with the linguistic finesse of LLaMA, fostering an intertwined learning process.

A pivotal challenge faced in this endeavor is the scarcity of data that link vision with language in an instruction-following format. LLaVA elegantly circumvents this by transforming conventional image–text pairs into this desired format, leveraging the capabilities of models like GPT-4.

Recent studies have highlighted the importance of the quality of instruction-following data in determining the efficacy of the resultant models. LLaVA stands as a testament to this, pioneering the incorporation of text-only models like GPT-4 to enrich datasets, facilitating detailed descriptions, question-answering sessions, and intricate reasoning exercises.

In summary, LLaVA encapsulates the essence of multi-modal AI, bridging the gap between vision and language, and is poised to redefine the frontiers of general-purpose AI assistants.

### 5.1. LLaVA-13b for Image Road Analysis

The realm of image road analysis has undergone a paradigm shift with the introduction of LLaVA-13b. Unlike traditional computer vision models that mainly focus on object detection and classification, LLaVA-13b, being a visual language model, integrates the understanding of context with visual features. When fed with real-time road images, LLaVA-13b can pinpoint road conditions, potential obstacles, safety concerns, and even subtle details like road quality or visibility conditions, as can be observed in Table 4 and Table 5 for two types of prompts, the first one placing emphasis on the road conditions and the second on real-time suggestions.

Such a nuanced analysis is paramount for assisted driving systems. For instance, identifying a pedestrian crossing from a distance, coupled with the contextual understanding that it is a school zone during school hours, can lead the system to be extra cautious and alert the driver well in advance.

The incorporation of these models into traffic forecasting and image road analysis signifies the convergence of language processing, computer vision, and data analytics. However, it is essential to understand the limitations. Real-time applications, especially those related to driving assistance, demand high reliability; while these models provide advanced insights, their predictions should be combined with traditional safety mechanisms and sensors in vehicular systems. Moreover, continuous model updates and training with diverse datasets can help in accommodating ever-changing road conditions and scenarios, ensuring the safety and efficiency of the driving experience.

### 5.2. Image Hashing and Retrieval with DL Features for Enhanced Multimodal Descriptions

The realm of visual understanding has seen significant advancements with the fusion of DL and vast image databases. By further harnessing these developments, we aim to build a robust image retrieval system [39], wherein the primary goal is to map novel visual inputs to their closest known representations in a pre-constructed database. This database, enriched with descriptions from multimodal large language models (LLMs) such as LLaVA, serves as a reservoir of insights that can be leveraged for real-time contextual understanding and instruction generation.

Consider a set $D=\{O_{1},O_{2},\ldots ,O_{N}\}$, representing *N* traffic images that serve as our reference database.

A standard preprocessing pipeline P(·) is defined as follows:

(12) $$O_{\mathrm{processed}}=P\left(O\right),$$

where P(·) encompasses operations like resizing, center cropping, tensor conversion, and normalization.

For extracting deep features, we employ a modified ResNet-50 architecture, denoted as R(·). The last classification layer of the pretrained ResNet-50 is excised to obtain a feature vector for any image:

(13) $$f_{o}=R\left(P\left(O_{o}\right)\right),$$

where $f_{o}\in R^{D}$ is the deep feature vector of the $o^{\mathrm{th}}$ image, and *D* is the feature dimensionality.

Our database is then represented as a collection of deep features:

(14) $$F=\{f_{1},f_{2},\cdots ,f_{N}\}.$$

Alongside each feature vector, we store enhanced descriptions generated by LLaVA, enabling rich semantic understanding.

Given a novel image $O_{\mathrm{sample}}$, its closest match from the database is identified using the Euclidean distance:

(15) $$o^{*}=\underset{o}{\mathrm{argmin}}\;{∥f_{\mathrm{sample}}-f_{o}∥}_{2},$$

where $f_{\mathrm{sample}}=R\left(P\left(O_{\mathrm{sample}}\right)\right)$.

**Figure 7:**
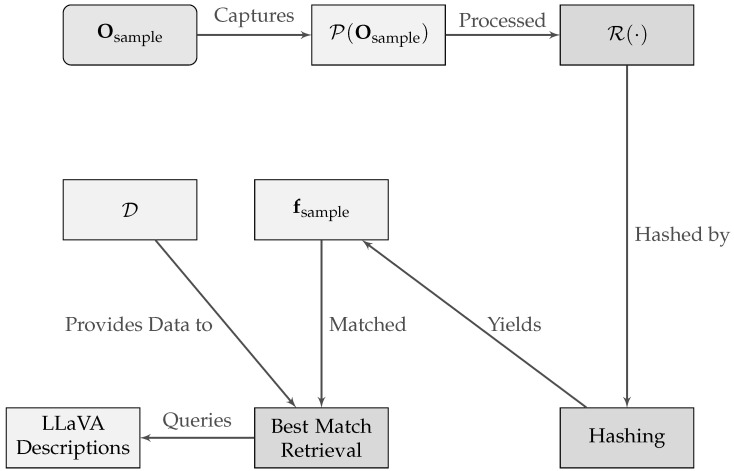
Diagrammatic representation of the image hashing and retrieval mechanism infused with DL and enriched by LLaVA.

Upon identifying the closest match, its associated LLaVA description is retrieved, offering additional semantic details of the visual scene in similar edge cases previously stored by the self-driving operators. This repository of LLaVA descriptions, constructed offline, ensures real-time efficiency and enables the system to offer enriched, context-aware instructions or insights to be combined with the real-time semantic information provided by compact LLMs such as LLaMA-2 and the actual LLaVA description for the sample. Figure 7 illustrates the procedure. Starting with a new image, $O_{\mathrm{sample}}$, the process follows a pipeline: it is first processed via P(·), then undergoes feature extraction using ResNet-50 denoted as R(·). The extracted features, $f_{\mathrm{sample}}$, are hashed and matched against an existing database, D. Upon finding the most similar image, enriched LLaVA descriptions from the database are retrieved to provide an augmented understanding of the image’s content and context in similar edge cases selected by the self-driving operators.

**Figure 8:**
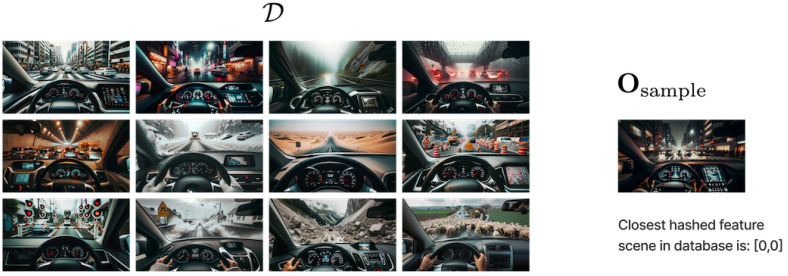
Illustrative representation of a sample database D consisting of 12 distinct traffic images representing edge cases selected by the self-driving operators.

Merging the power of DL-based image hashing with multimodal LLMs presents a promising paradigm for real-time visual understanding. By tapping into pre-generated LLaVA descriptions, our system efficiently contextualizes novel visual inputs, promoting real-time, informed decision making in dynamic urban environments. An illustrative example can be seen in Figure 8, where the queried sample, $O_{\mathrm{sample}}$, after undergoing the proposed hashing and retrieval mechanism, yields a closest hashed feature vector of [0,0], signifying its high similarity to one of the pre-existing samples in D, from which we select its corresponding LLaVA description for further additional information to add to a compact LLM such as LLaMA-2 and the actual LLaVA description for the sample.

### 5.3. Bayesian Image Feature Extraction and Uncertainty Analysis

The evolution of computer vision has been critically influenced by the integration of Bayesian methodologies. In contrast to conventional deterministic models, Bayesian models proffer not only point estimates but also uncertainties tied to those estimates. This subsection elucidates an avant-garde methodology that employs a Bayesian version of the ResNet model for deep feature extraction, accompanied by an analysis of the uncertainties inherent to the derived features with the use of the deep probabilistic framework Pyro [18,40].

#### 5.3.1. Bayesian Adaptation of ResNet

The following phase of our approach transforms the canonical ResNet architecture into its Bayesian analogue. This transformation is key, infusing probabilistic layers into the model’s linear layers, permitting it to encompass uncertainties within the feature space. Specifically, each parameter (weights and biases) in these layers is perceived as a sample from a Gaussian distribution, enabling the model to produce an ensemble of feature outputs for any given image, rather than a unique deterministic output.

The Bayesian layer can be mathematically expressed as follows:

(16) $$\begin{array}{cc} w & ∼N(0,I), \end{array}$$

(17) $$\begin{array}{cc} b & ∼N(0,I), \end{array}$$

where w and b denote the weight and bias matrices, respectively.

#### 5.3.2. Feature Extraction and Image Hashing

The subsequent phase involves a streamlined preprocessing mechanism, manifesting every image as $O_{\mathrm{processed}}=P\left(O\right)$. After preprocessing, as images are streamed through the Bayesian ResNet, an ensemble of feature vectors emerge due to the model’s probabilistic nature. From this ensemble, two cardinal statistics, the mean μ and variance $\sigma^{2}$, are derived:

(18) $$\mu =\frac{1}{M}\sum_{o=1}^{M}R\left(O_{\mathrm{processed}}^{\left(o\right)}\right),\;\sigma^{2}=\frac{1}{M}\sum_{o=1}^{M}{\left(R(O_{\mathrm{processed}}^{\left(o\right)})-\mu \right)}^{2},$$

where *M* is the number of forward passes through the Bayesian ResNet.

#### 5.3.3. Uncertainty Analysis

The variance, representing the spread of the feature values, serves as a quantifier of uncertainty. This uncertainty provides the following:

- Insights into dimensions bearing both maximal and minimal confidence.
- Potential triggers for precautionary measures in applications demanding high reliability, facilitating a more transparent and robust system.

Incorporating Bayesian frameworks within the realm of image processing augments the field in a twofold manner: by extracting deep probabilistic feature representations encapsulating an image’s close resemblances and by quantifying the uncertainty tethered to these features. This fusion of deterministic insights with probabilistic interpretations paves the path for more resilient and transparent image interpretation mechanisms. Prospective endeavors could expand this technique across diverse architectures and gauge its efficacy in high-stakes, real-time scenarios.

**Figure 9:**
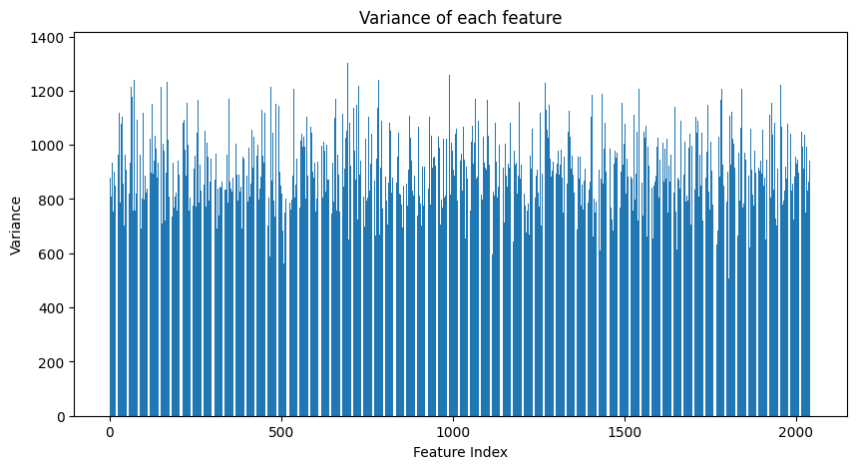
Histogram illustrating the variance of each feature derived from the Bayesian ResNet. Each bar represents the variance of a specific feature, highlighting the spread and uncertainty in the feature representations. The horizontal axis enumerates the features, while the vertical axis quantifies the variance, emphasizing regions of high and low confidence in the extracted features.

In our particular illustrative case, see Figure 8, the closest matching scene in database depicts that, based on the deep features extracted from the Bayesian model, the sample image is most similar to the image [0,0] in the reference database. The output of the system is as follows:

- Mean features: This is the average value of the deep features for the sample after passing it through the Bayesian model multiple times. The deep features give a high-level, abstract representation of the image, and the mean provides the “central” or “most likely” representation in the presence of uncertainties in the model.
- Variance in features (see Figure 9): This captures the uncertainty or variability in the deep features for the sample under study after multiple forward passes through the Bayesian model. Higher values of variance for certain feature dimensions indicate higher uncertainties in those dimensions. This could arise from various factors like the inherent noise in the image, ambiguities in the scene, or uncertainties in the model parameters.

Interpretation:

For image matching, the user can infer that these images are potentially of similar scenes or contain similar objects/structures.

For uncertainty analysis: The mean and variance provide a way to understand not just what the model “thinks” but how “confident” it is in its thinking. Dimensions with high variance indicate that there is significant uncertainty about that particular aspect of the image’s representation. This can be particularly useful in scenarios where the system has to act conservatively or alert a human operator in the presence of high uncertainty.

For instance, in safety-critical applications like in the application of self-driving cars, if certain features of a new scene (like the position of a pedestrian) have high variance, the system might decide to slow down or take a precautionary measure.

In summary, while the closest matching image provides actionable insight into the scene, the variance in the deep features provides a measure of confidence or reliability about the derived insights, making the system more robust and transparent and quantifiable.

In the pursuit of advancing autonomous driving systems, various methodologies have been employed, each serving distinct purposes and contributing uniquely to the overarching goal of enhanced safety and efficiency. To provide a clear and concise comparison of these methodologies, we present in Table 6 a summary that delineates their respective applications, advantages, disadvantages, and limitations in practical scenarios. This comparison aims to offer insights into how each method complements the others, while also highlighting areas that may require attention or further improvement. The table encompasses a range of techniques from DL forecasting and dimensionality reduction to the utilization of LLMs and Bayesian image feature extraction, all of which play fundamental roles in the real-time assessment and intervention for safer autonomous driving.

Regarding the data used in the experiments, specific datasets have been utilized for different aspects of our methodologies. For the study of PCA loadings, as well as for the training and validation of our Transformer-based models, we have leveraged the Fatality Analysis Reporting System (FARS) dataset from the year 2020. FARS2020 is a nationwide database, curated by the National Highway Traffic Safety Administration (NHTSA), which encapsulates a wide array of information pertaining to road traffic accidents, including details about the vehicles involved, the geographical and temporal aspects of the incidents, and the associated human factors, as described earlier in the manuscript.

On the visual front, for the evaluation of our VLMs and Bayesian feature extraction, we have employed a set of generative samples from a mock self-driving simulator created using DALL-E 3 [41]. These images depict a variety of scenarios that a self-driving vehicle might encounter, ranging from common urban and rural driving conditions to more challenging and adverse weather situations like rain, snow, and fog.

## 6. Discussion

This study marks a significant advancement in the field of autonomous driving by introducing a novel approach to influence the driving style of L-4 and L-5 autonomous vehicles. Utilizing LLMs and VLMs, informed by PCA loadings from extensive historical traffic data, we have established a method that bridges the gap between traditional and modern autonomous driving techniques. Unlike existing methodologies that predominantly rely on LiDAR and hard-coded guidelines, our approach ensures a more adaptive and context-aware decision-making process, integrating both historical patterns and real-time visual road analysis.

The utilization of PCA loadings as feature weights plays a crucial role in providing the LLMs with a rich context for making informed decisions, reflecting the true nature of the driving environment. This incorporation of historical data enhances the vehicle’s ability to anticipate and respond to potential hazards, thereby contributing to safer and more efficient driving practices. The real-time image road analysis provided by VLMs further ensures that the vehicle is aware of its immediate surroundings, allowing for quick and accurate adjustments to its driving style.

However, it is important to acknowledge the limitations of our approach. The reliance on comprehensive historical data and the need for accurate real-time image analysis pose challenges that must be addressed in future work. The system’s performance is contingent on the quality and accuracy of the data it receives, emphasizing the need for robust data collection and processing methods.

Our method introduces a novel approach to real-time accident prediction and driving behavior adjustment for autonomous vehicles, leveraging the synergies between PCA loadings, Transformer predictions, and LLMs. We acknowledge that this is a pioneering field, and direct comparisons might be challenging due to the uniqueness of our approach. However, we have ensured rigorous testing and validation of our method to showcase its effectiveness and reliability. In our experiments, we demonstrated that our method could successfully interpret and act upon potentially complex road scenarios, providing accurate and actionable insights. The integration of visual and textual data, processed through advanced ML models, ensures a comprehensive understanding of the driving environment, which is crucial for autonomous vehicles.

As a summary, this paper presents an innovative approach to enhancing autonomous driving through the integration of LLMs and VLMs, leveraging PCA loadings from historical traffic data. Our method demonstrates the potential of combining DL models with historical patterns and real-time visual cues, aiming to create a safer, more adaptive, and intelligent driving experience. This work lays a solid foundation for future research and development in the field of autonomous driving, opening new avenues for enhancing the capabilities of autonomous vehicles and ensuring their safe integration into our transportation systems.

## 7. Conclusions

This research elucidates the powerful synergy between PCA loadings (feature weights corresponding to the principal components), Transformer predictions, and leading-edge large language models (LLMs) such as LLaMA2, GPT-4, or Zephyr-7b-α. Specifically, our findings accentuate that, while each component offers substantial predictive insights, their combination provides a paradigm shift in how we approach accident forecasting. The semiotic richness of the LLMs not only refines our forecasts but also avails discernible, actionable insights, fundamental for on-ground implementation and real-time use.

Furthermore, the incorporation of visual language models (VLMs)—exemplified by LLaVA in our study—in conjunction with deep probabilistic reasoning introduces a novel dimension. This visual–semantic fusion equips our methodology with a holistic comprehension, enabling a nuanced understanding of road scenarios and accident predispositions.

In essence, the marriage of numerical, textual, and visual data, harnessed through advanced models, presents the vanguard of accident forecasting. It paves the way for safer urban landscapes, driven by enriched, multi-faceted data insights.

In highlighting the capabilities of our applied models, it is essential to mention the actionable dimensions of LLMs and VLMs in the realm of autonomous driving. Our research showcases how multimodal LLMs can translate complex data predictions into pragmatic strategies for self-driving vehicles. By inferring real-time information, these models offer a proactive approach to adjusting driving strategies based on evolving road conditions, traffic patterns, and potential hazards. Furthermore, the framework presents a methodology for extracting probabilistic edge-case scenarios from past databases, thereby enhancing a vehicle’s risk assessment capabilities. In essence, while the presented LLMs may not directly "act", their outputs certainly inform and shape the actions of autonomous systems, providing them with a robust decision-making foundation. This active learning and adaptation ability positions large multimodal models as a foundation for future developments in autonomous transportation, ensuring safer and more informed vehicular operations.

## Figures and Tables

**Table 1:** PCA loadings 1–6.

Component	Loadings
Component 1	FUNC_SYS_96: Trafficway Not in State Inventory, RUR_URB_6: Trafficway Not in State Inventory, LGT_COND_5: Dusk, DAY_15: Day of Crash—The accident occurred on the 15th day of the month, HOUR_20: Hour of Crash—The accident occurred at 8 p.m., MONTH_8: Month of Crash—The accident occurred in August, WEATHER_10: Cloudy, PEDS_1: Number of Persons Not in Motor Vehicles—one person, LGT_COND_9: Reported as Unknown, DRUNK_DR_1: Number of Drinking Drivers Involved in the Fatal Crash—one driver.
Component 2	VE_TOTAL_2: Number of Vehicles in Crash—two vehicles, PERSONS_5: Number of Person Forms—five persons, LGT_COND_3: Dark but Lighted, FATALS_4: Number of Fatalities That Occurred in the Crash—four fatalities, PERSONS_2: Number of Person Forms—two persons, WEATHER_5: Fog, Smog, Smoke, PEDS_1: Number of Persons Not in Motor Vehicles—one person, DAY_5: Day of Crash—The accident occurred on the fifth day of the month, RUR_URB_9: Land Use—Unknown, FUNC_SYS_99: Functional System—Unknown.
Component 3	FUNC_SYS_99: Functional System—Unknown, RUR_URB_9: Land Use—Unknown, FATALS_4: Number of Fatalities That Occurred in the Crash—four fatalities, DAY_28: Day of Crash—The accident occurred on the 28th day of the month, HOUR_9: Hour of Crash—The accident occurred at 9 a.m., WEATHER_5: Fog, Smog, Smoke, HOUR_2: Hour of Crash—The accident occurred at 2 a.m., DAY_5: Day of Crash—The accident occurred on the fifth day of the month, DAY_4: Day of Crash—The accident occurred on the fourth day of the month, HOUR_17: Hour of Crash—The accident occurred at 5 p.m.
Component 4	LGT_COND_3: Dark but Lighted, HOUR_7: Hour of Crash—The accident occurred at 7 a.m., DAY_31: Day of Crash—The accident occurred on the 31st day of the month, RUR_URB_9: Land Use—Unknown, HOUR_23: Hour of Crash—The accident occurred at 11 p.m., HOUR_0: Hour of Crash—The accident occurred at 12 a.m., WEATHER_5: Fog, Smog, Smoke, MONTH_2: Month of Crash—The accident occurred in February, LGT_COND_2: Dark—Not Lighted, DAY_6: Day of Crash—The accident occurred on the sixth day of the month.
Component 5	HOUR_17: Hour of Crash—The accident occurred at 5 p.m., LGT_COND_2: Dark—Not Lighted, HOUR_18: Hour of Crash—The accident occurred at 6 p.m., PERSONS_2: Number of Person Forms—two persons, VE_TOTAL_2: Number of Vehicles in Crash—two vehicles, HOUR_3: Hour of Crash—The accident occurred at 3 a.m., HOUR_6: Hour of Crash—The accident occurred at 6 a.m., DAY_14: Day of Crash—The accident occurred on the 14th day of the month, FATALS_2: Number of Fatalities That Occurred in the Crash—two fatalities, PERSONS_1: Number of Person Forms—one person.
Component 6	HOUR_6: Hour of Crash—The accident occurred at 6 a.m., LGT_COND_2: Dark—Not Lighted, HOUR_7: Hour of Crash—The accident occurred at 7 a.m., RUR_URB_9: Land Use—Unknown, LGT_COND_5: Dusk, HOUR_5: Hour of Crash—The accident occurred at 5 a.m., LGT_COND_3: Dark but Lighted, PERSONS_2: Number of Person Forms—two persons, VE_TOTAL_2: Number of Vehicles in Crash—two vehicles, DAY_30: Day of Crash—The accident occurred on the 30th day of the month.

**Table 2:** PCA loadings 7–11.

Component	Loadings
Component 7	LGT_COND_3: Dark but Lighted, LGT_COND_2: Dark—Not Lighted, LGT_COND_5: Dusk, HOUR_0: Hour of Crash—The accident occurred at 12 a.m., HOUR_7: Hour of Crash—The accident occurred at 7 a.m., HOUR_23: Hour of Crash—The accident occurred at 11 p.m., DAY_1: Day of Crash—The accident occurred on the first day of the month, DAY_2: Day of Crash—The accident occurred on the second day of the month, RUR_URB_9: Land Use—Unknown, DAY_3: Day of Crash—The accident occurred on the third day of the month.
Component 8	HOUR_7: Hour of Crash—The accident occurred at 7 a.m., HOUR_6: Hour of Crash—The accident occurred at 6 a.m., LGT_COND_2: Dark—Not Lighted, LGT_COND_3: Dark but Lighted, HOUR_8: Hour of Crash—The accident occurred at 8 a.m., DAY_1: Day of Crash—The accident occurred on the first day of the month, DAY_2: Day of Crash—The accident occurred on the second day of the month, PERSONS_2: Number of Person Forms—two persons, VE_TOTAL_2: Number of Vehicles in Crash—two vehicles, LGT_COND_5: Dusk.
Component 9	LGT_COND_3: Dark but Lighted, LGT_COND_2: Dark—Not Lighted, RUR_URB_9: Land Use—Unknown, HOUR_6: Hour of Crash—The accident occurred at 6 a.m., HOUR_7: Hour of Crash—The accident occurred at 7 a.m., DAY_1: Day of Crash—The accident occurred on the first day of the month, LGT_COND_5: Dusk, HOUR_8: Hour of Crash—The accident occurred at 8 a.m., HOUR_5: Hour of Crash—The accident occurred at 5 a.m., PERSONS_2: Number of Person Forms—two persons.
Component 10	HOUR_8: Hour of Crash—The accident occurred at 8 a.m., LGT_COND_2: Dark—Not Lighted, LGT_COND_3: Dark but Lighted, HOUR_7: Hour of Crash—The accident occurred at 7 a.m., RUR_URB_9: Land Use—Unknown, HOUR_6: Hour of Crash—The accident occurred at 6 a.m., DAY_1: Day of Crash—The accident occurred on the first day of the month, LGT_COND_5: Dusk, PERSONS_2: Number of Person Forms—two persons, VE_TOTAL_2: Number of Vehicles in Crash—two vehicles.
Component 11	LGT_COND_2: Dark—Not Lighted, LGT_COND_3: Dark but Lighted, HOUR_8: Hour of Crash—The accident occurred at 8 a.m., HOUR_7: Hour of Crash—The accident occurred at 7 a.m., RUR_URB_9: Land Use—Unknown, DAY_1: Day of Crash—The accident occurred on the first day of the month, HOUR_6: Hour of Crash—The accident occurred at 6 a.m., PERSONS_2: Number of Person Forms—two persons, VE_TOTAL_2: Number of Vehicles in Crash—two vehicles, LGT_COND_5: Dusk.

**Table 3:** Summary of PCA loadings 1–11.

Component	Primary Influences
Component 1	This component is strongly influenced by variables such as the functional system of the roadway (with a specific emphasis on trafficways not in the state inventory), the land use context indicating trafficways not in the state inventory, light conditions during the accident (specifically, unknown lighting conditions), and the presence of alcohol in drivers.
Component 2	The second component focuses on the total number of vehicles involved in the crash, the number of persons involved, light conditions at dawn, the number of fatalities, and the type of weather conditions (cloudy).
Component 3	This component is influenced by the functional system of the roadway (unknown category), land use context indicating unknown urbanization, the number of fatalities, and specific days and hours during which the accident occurred.
Component 4	The fourth component places emphasis on the light conditions during the accident (specifically, unknown lighting conditions), rural or urban context of the accident site, and the functional system of the roadway.
Component 5	This component is influenced by the month of the accident, the number of persons involved, and the land use context, specifically differentiating between urban and rural environments.
Component 6	The sixth component seems to be influenced mainly by the total number of vehicles involved in the crash, the functional system of the roadway (especially interstate roads), and the number of persons involved in the accident.
Component 7	This component is influenced by the functional system of the roadway, the number of vehicles involved in the crash, and the specific days and hours during which the accident occurred.
Component 8	The eighth component emphasizes the functional system of the roadway, the number of persons not in motor vehicles, and specific weather conditions during the accident.
Component 9	This component focuses on the number of persons involved in the accident, specific days during which the accident occurred, and the functional system of the roadway.
Component 10	The tenth component places emphasis on the functional system of the roadway, specific days during which the accident occurred, and the hour of the crash.
Component 11	This component seems to be influenced mainly by the number of persons not in motor vehicles during the accident, the land use context indicating the urbanization of the accident site, and the functional system of the roadway.

**Table 4:** Analysis of road conditions using LLaVA.

Sample	Prompt: Analyze the Road Conditions, Potential Obstacles, and Safety Concerns Visible in This Image.
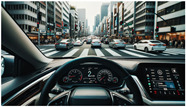	In the image, we see a busy city street with a large number of cars and pedestrians. The road is filled with traffic, including cars, trucks, and even a bus. There are multiple traffic lights along the street, which helps manage the flow of vehicles and pedestrians. The potential obstacles and safety concerns visible in the image include the high volume of traffic, which can lead to congestion and increased risk of accidents. The presence of pedestrians crossing the street also adds to the complexity of the scene, as drivers must be vigilant and cautious to avoid collisions. Additionally, the image shows a car driving through an intersection, which is a common area for accidents to occur. Drivers must pay close attention to the traffic lights and other vehicles to ensure safe navigation through the intersection. Overall, the image depicts a bustling city street with various obstacles and safety concerns that require drivers and pedestrians to be attentive and cautious to maintain safety and order.
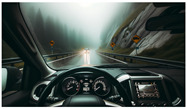	In the image, the road appears to be wet, which can lead to reduced visibility and potentially slippery driving conditions. The car’s headlights are on, indicating that the driver is trying to improve visibility in the foggy and wet conditions. However, the foggy conditions can still make it challenging for the driver to see clearly and anticipate potential obstacles. There are several traffic signs visible along the road, which are essential for providing guidance and information to drivers. The presence of these signs indicates that the road is well-maintained and organized, reducing the risk of accidents. The image also shows a car driving down the road, which could be a potential obstacle for other vehicles. Drivers should be cautious and maintain a safe distance from other vehicles to avoid collisions in such conditions. Overall, the image highlights the importance of being vigilant and cautious while driving in wet and foggy conditions, as visibility is reduced, and the risk of accidents can increase.

**Table 5:** Real-time analysis of road conditions using LLaVA.

Sample	Prompt: Provide a Real-Time Analysis of the Road Conditions, Potential Obstacles, and Safety Concerns Visible in This Image. Update on Any Changes or Movements That Might Affect Driving Decisions.
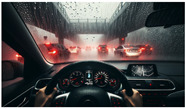	In the image, a car is driving down a wet highway with heavy rain. The road appears to be wet, which can lead to reduced visibility and increased braking distance. The driver should exercise caution and maintain a safe distance from other vehicles. There are several cars on the road, including a truck, which indicates that the traffic is moderate. The driver should be aware of other vehicles’ movements and adjust their speed accordingly to avoid collisions. Additionally, the presence of traffic lights suggests that the driver should pay attention to the signals and follow the rules of the road. Overall, the driver should remain vigilant and cautious while driving in these conditions, as the wet road and heavy rain can pose challenges to safe driving.
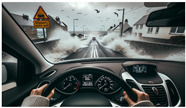	In the image, a car is driving down a road near a beach, with a surreal scene of a wave crashing into the beach and a flock of birds flying overhead. The road appears to be wet, which could indicate recent rain or a nearby water source. The presence of birds flying close to the road could pose a potential hazard for the driver, as they might suddenly land on the road or distract the driver. The surreal wave crashing into the beach suggests that the area might be prone to flooding or other natural disasters, which could impact driving conditions and safety. The driver should remain vigilant and cautious while navigating the road, taking into account the wet road conditions, potential bird hazards, and the possibility of natural disasters.

**Table 6:** Comparison of used methods.

Method	Application	Advantages	Disadvantages	Limits in Practice
DL	Time series forecasting of traffic data	High accuracy, captures complex patterns	Requires large amounts of data, computationally expensive	May not perform well with sparse or noisy data
PCA	Dimensionality reduction for traffic data	Reduces data complexity, helps identify major patterns	Loss of information, assumes linear relationships	Not suitable for non-linear patterns
LLMs	Interpreting PCA loadings, real-time traffic forecasting, real-time risk assessment	High interpretability, versatile applications	Requires large computational resources, may generate biased outputs	May not always provide accurate real-time responses
LLaVA-13b, GPT-4V	Detailed image road analysis	High accuracy, provides context-aware insights	Computationally expensive, requires large amounts of data	May struggle with ambiguous or novel scenarios
Bayesian image feature extraction	Deep feature extraction with uncertainty analysis	Provides uncertainty estimates, enhances robustness	Computationally expensive, requires understanding of Bayesian methods	May be overkill for simple applications
Real-time intervention	Dynamic adjustment of driving style based on risk assessment	Enhances vehicle safety, adaptable to changing conditions	Depends on accurate risk assessment, may require frequent updates	May not cover all potential road scenarios

## Data Availability

The data presented in this study are openly available in FigShare at https://doi.org/10.6084/m9.figshare.24570478.

## Abbreviations

The following abbreviations are used in this manuscript:

Large Language Models	LLMs
Visual Language Models	VLMs
Principal Component Analysis	PCA
Generative Pre-Trained Transformers 4 with Vision	GPT-4V
Deep Learning	DL
Machine Learning	ML
Artificial Intelligence	AI
Large Language Model Meta AI	LLaMA
Large Language and Vision Assistant	LLaVA
AutoRegressive Integrated Moving Average	ARIMA
